# A steeply-inclined trajectory for the Chicxulub impact

**DOI:** 10.1038/s41467-020-15269-x

**Published:** 2020-05-26

**Authors:** G. S. Collins, N. Patel, T. M. Davison, A. S. P. Rae, J. V. Morgan, S. P. S. Gulick, IODP-ICDP Expedition 364 Science Party, G. L. Christeson, E. Chenot, P. Claeys, C. S. Cockell, M. J. L. Coolen, L. Ferrière, C. Gebhardt, K. Goto, H. Jones, D. A. Kring, J. Lofi, C. M. Lowery, R. Ocampo-Torres, L. Perez-Cruz, A. E. Pickersgill, M. H. Poelchau, C. Rasmussen, M. Rebolledo-Vieyra, U. Riller, H. Sato, J. Smit, S. M. Tikoo, N. Tomioka, J. Urrutia-Fucugauchi, M. T. Whalen, A. Wittmann, L. Xiao, K. E. Yamaguchi, Third-Party Scientists, N. Artemieva, T. J. Bralower

**Affiliations:** 1 Department Earth Science and Engineering, Imperial College London, London, SW7 2AZ UK; 2 Institute of Geology, University of Freiburg, Freiburg, 79104 Germany; 3 Institute for Geophysics and Department of Geological Sciences, University of Texas at Austin, Austin, TX 78758 USA; 4 Institute for Geophysics, University of Texas at Austin, Austin, TX USA; 5 Laboratoire GeoRessources, Université de Lorraine, Vandoeuvre-lés-Nancy, France; 6 Analytical, Environmental and Geochemistry, Vrije Universiteit Brussel, Brussels, Belgium; 7 UK Centre for Astrobiology, School of Physics and Astronomy, University of Edinburgh, Edinburgh, UK; 8 Western Australia Organic and Isotope Geochemistry Centre, School of Earth and Planetary Sciences, Curtin University, Bentley, WA 6102 Australia; 9 Natural History Museum, Vienna, Austria; 10 Alfred Wegener Institute Helmholtz Centre of Polar and Marine Research, Bremerhaven, Germany; 11 Department of Earth and Planetary Science, University of Tokyo, Tokyo, Japan; 12 Department of Geosciences, Pennsylvania State University, University Park, PA USA; 13 Lunar and Planetary Institute, Houston, TX USA; 14 Géosciences Montpellier, CNRS, Université de Montpellier, Montpellier, France; 15 Groupe de Physico-Chimie de l’Atmosphère, L’Institut de Chimie et Procédés pour l’Énergie, l’Environnement et la Santé, Université de Strasbourg, Strasbourg, France; 16 Instituto de Geofísica, Universidad Nacional Autónoma De México, Ciudad De México, Mexico; 17 School of Geographical and Earth Sciences, University of Glasgow, Glasgow, UK; 18 Argon Isotope Facility, Scottish Universities Environmental Research Centre, East Kilbride, UK; 19 Department of Geology and Geophysics, University of Utah, Salt Lake City, UT USA; 20 Unidad de Ciencias del Agua, Mérida, Mexico; 21 Institut für Geologie, Universität Hamburg, Hamburg, Germany; 22 Japan Agency for Marine-Earth Science and Technology, Kanagawa, Japan; 23 Faculty of Earth and Life Sciences, Vrije Universiteit Amsterdam, Amsterdam, Netherlands; 24 Department of Earth and Planetary Sciences, Rutgers University, Piscataway Township, NJ USA; 25 Department of Geophysics, Stanford University, Stanford, CA USA; 26 Kochi Institute for Core Sample Research, Japan Agency for Marine-Earth Science and Technology, Kochi, Japan; 27 Department of Geosciences, University of Alaska Fairbanks, Fairbanks, AK USA; 28 Eyring Materials Center, Arizona State University, Tempe, AZ USA; 29 School of Earth Sciences, Planetary Science Institute, China University of Geosciences, Wuhan, China; 30 Department of Chemistry, Toho University, Funabashi, Chiba Japan; 31 NASA Astrobiology Institute, Mountain View, CA USA; 32 Planetary Science Institute, Tucson, AZ USA

**Keywords:** Asteroids, comets and Kuiper belt, Asteroids, comets and Kuiper belt

## Abstract

The environmental severity of large impacts on Earth is influenced by their impact trajectory. Impact direction and angle to the target plane affect the volume and depth of origin of vaporized target, as well as the trajectories of ejected material. The asteroid impact that formed the 66 Ma Chicxulub crater had a profound and catastrophic effect on Earth’s environment, but the impact trajectory is debated. Here we show that impact angle and direction can be diagnosed by asymmetries in the subsurface structure of the Chicxulub crater. Comparison of 3D numerical simulations of Chicxulub-scale impacts with geophysical observations suggests that the Chicxulub crater was formed by a steeply-inclined (45–60° to horizontal) impact from the northeast; several lines of evidence rule out a low angle (<30°) impact. A steeply-inclined impact produces a nearly symmetric distribution of ejected rock and releases more climate-changing gases per impactor mass than either a very shallow or near-vertical impact.

## Abstract (web summary)

The authors here present a 3D model that simulates the formation of the Chicxulub impact crater. Based on asymmetries in the subsurface structure of the Chicxulub crater, the authors diagnose impact angle and direction and suggest a steeply inclined (60° to horizontal) impact from the northeast.

## Introduction

The 66 Ma asteroid impact event that formed the Chicxulub crater, Mexico, marks the end of the Mesozoic Era of Earth history and has been attributed as the cause of the contemporaneous mass extinction^1^. Numerical impact simulations combined with geophysical investigation of subsurface structure have constrained the kinetic energy of the impact under the simplifying assumption of a vertical trajectory^2–4^. The trajectory angle and direction of the Chicxulub impact are not known, but a near-vertical impact is unlikely. Only one quarter of impacts occur at angles between $6{0}^{\circ }$ and the vertical and only 1 in 15 impacts is steeper than $7{5}^{\circ }$.

For constant impactor size and speed, a shallower impact angle produces a smaller crater, a more asymmetric dispersal of ejected material^5^ and partitions more impact energy at shallower depths^6^. As a result, impact direction and trajectory angle to the target plane are important impact parameters that determine, among other things, the direction of most severe environmental consequences and the volume and depth of origin of vaporised target^7,8^, as well as ejecta^5^ and crater asymmetries^9^.

**Fig. 1:**
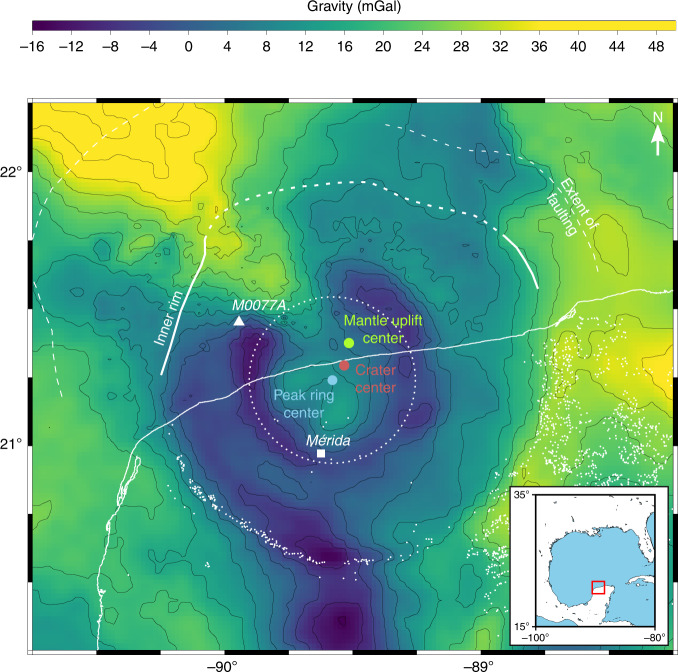
Asymmetries of the geophysical signature of the Chicxulub crater. Background colourmap shows Bouguer gravity anomaly map in the vicinity of the crater (gravity data courtesy of Hildebrand and Pilkington). The red circle marks the nominal position of the crater centre; the green circle marks the centre of maximum mantle uplift; the blue circle marks the centre of the peak ring (as defined by the annular gravity low surrounding the central high); the white triangle marks the location of the Expedition 364 drill site through the peak ring (Hole M0077A). The coastline is displayed with a thin white line; cenotes and sinkholes with white dots, and the city of Mérida with a white square. The dotted lines offshore mark the approximate location of the inner crater rim and the extent of faulting as imaged by seismic data^14^. Inset depicts the regional setting, with red rectangle outlining the region shown in the gravity map. Adapted from ref. ^14^.

Since the discovery of the Chicxulub impact structure based on diagnostic evidence of shock metamorphism and geophysical anomalies^10^, several asymmetries in the geophysical character of the crater have been noted^8,11,12^, which may result from oblique impact^8,11^ and/or impact in a heterogeneous target^3,12^. Among the most obvious of these (Fig. 1) are radially oriented gravity lows to the south and northeast, and a radial gravity high to the northwest^8,13^. However, these large-scale, peripheral features are all likely to be pre-existing features, unrelated to the impact^12^. As models of the subsurface based on potential field data have inherently poor resolution and suffer from non-uniqueness, the most robust evidence of asymmetry comes from seismic reflection and refraction data^14^. High-resolution seismic reflection images along a concentric arc outside the crater rim clearly show that the northeastern gravity low in the offshore half of the crater occurs in an area where Cretaceous and Cenozoic sedimentary rocks are particularly thick, and the northwestern gravity high occurs where this sedimentary sequence is thinnest and basement rocks are closer to surface (ref. ^3^, Fig. 3). Given the observed correlation between the gravity signature and depth to basement outside the crater, the thickness of the sedimentary sequence is the most likely control on the offshore gravity anomaly. There is therefore no evidence that the azimuthal asymmetry in the outer gravity signature is impact related.

A nominal geographical centre of the crater (21.29° N, 89.53° W) is defined by the geometric centre of the crater rim demarcated by both a circular high in horizontal gravity gradient and the prominent cenote ring^11,13^ (Fig. 1). Relative to this point, the centre (21.24° N, 89.58° W) of both the central gravity high, attributed to the uplift of dense lower-crustal rocks^11^, and the surrounding annular gravity low, which underlies the inner edge of the peak ring, are shifted several km to the west-southwest (Fig. 1 and Supplementary Fig. 1). In contrast, three-dimensional (3D) seismic velocity data indicate that the maximum uplift of the mantle beneath the crater occurs at 21.38° N, 89.52° W, ca. 10 km to the north-northeast of the crater centre^4^ (Fig. 1, Supplementary Fig. 1 and ref. ^4^, Fig. 4d).

The south-westerly offset of the central gravity high relative to the crater centre was previously interpreted as indicating impact from the southwest, on the premise that central uplift motion would be directed uprange^11^. An alternative interpretation, of a trajectory from the southeast, was proposed on the basis of a northwest–southeast elongation of the central gravity high and magnetic anomaly, and the northwest truncation of the annular gravity low^8^. However, seismic reflection and refraction data reveal that the zone of structural uplift is not elongated towards the northwest^14^ (see also Supplementary Fig. 1) and that the truncation of the gravity low in the northwest is a pre-impact feature of the regional anomaly caused by the shallow depth to basement in this direction. The short-wavelength component of the magnetic anomaly shows a slight (10%) elongation in the northwest–southeast direction^15,16^, but is also offset to the southwest of the crater centre (Supplementary Fig. 1). The short wavelength and steep gradients of this anomaly both suggest a shallow source, probably related to the melt sheet and impact breccia and not to structural crater asymmetry^15,16^. On the other hand, the long-wavelength component of the magnetic anomaly is a magnetic high elongated and offset along a direction southwest of the crater centre (Supplementary Fig. 1), consistent with a zone of uplifted basement rocks southwest of the crater centre^15^.

Here we use 3D numerical modelling to examine the relationship between impact angle and structural crater asymmetries in a Chicxulub-scale peak-ring crater in a flat-layered target without lateral pre-impact asymmetry. We show that the observed asymmetry in the positions of the central uplift, peak-ring centre and maximum mantle uplift, relative to the crater centre, can be attributed to the angle and azimuth of the impact trajectory. Comparison of our simulation results with geophysically constrained models of the Chicxulub crater structure is used to infer the likely trajectory and angle of the impact. The recent joint International Ocean Discovery Program (IODP) and International Continental Scientific Drilling Program (ICDP) Expedition 364 recovered ~600 m of peak-ring rocks from the Chicxulub crater^17^ that provide additional constraints to discriminate between impact scenarios. Our simulations also reveal azimuthal variation in peak-ring material properties, which provide context for IODP-ICDP Expedition 364 core analysis.

## Results and discussion

### Numerical simulation results

**Fig. 2:**
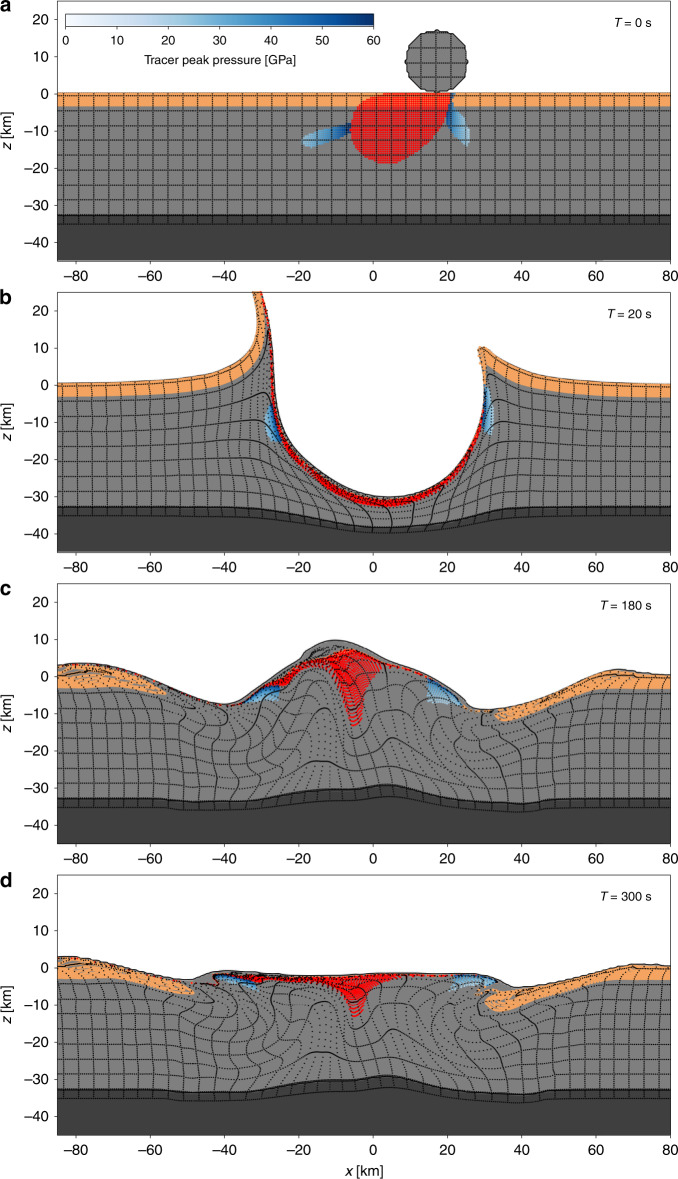
Development of the Chicxulub crater for a $6{0}^{\circ }$ impact. The impact scenario depicted is for a 17-km diameter impactor with a density of 2630 kg m^3^ and a speed of 12 km/s. Evolution of the crater up to 5 min after impact is depicted. Shown are cross-sections through the numerical simulation along the plane of trajectory, with $x=0$ defined at the crater centre (measured at the pre-impact level; $z=0$); the direction of impact is from right to left. The upper 3 km of the pre-impact target, corresponding to the average thickness of sedimentary rocks at Chicxulub, is tracked by tracer particles (sandy brown). Deformation in the crust (mid-grey) and upper mantle (dark grey) is depicted by a grid of tracer particles (black). Tracer particles within the peak-ring material are highlighted based on the peak shock pressure recorded (white–blue colour scale); melted target material (>60 GPa) is highlighted in red.

**Fig. 3:**
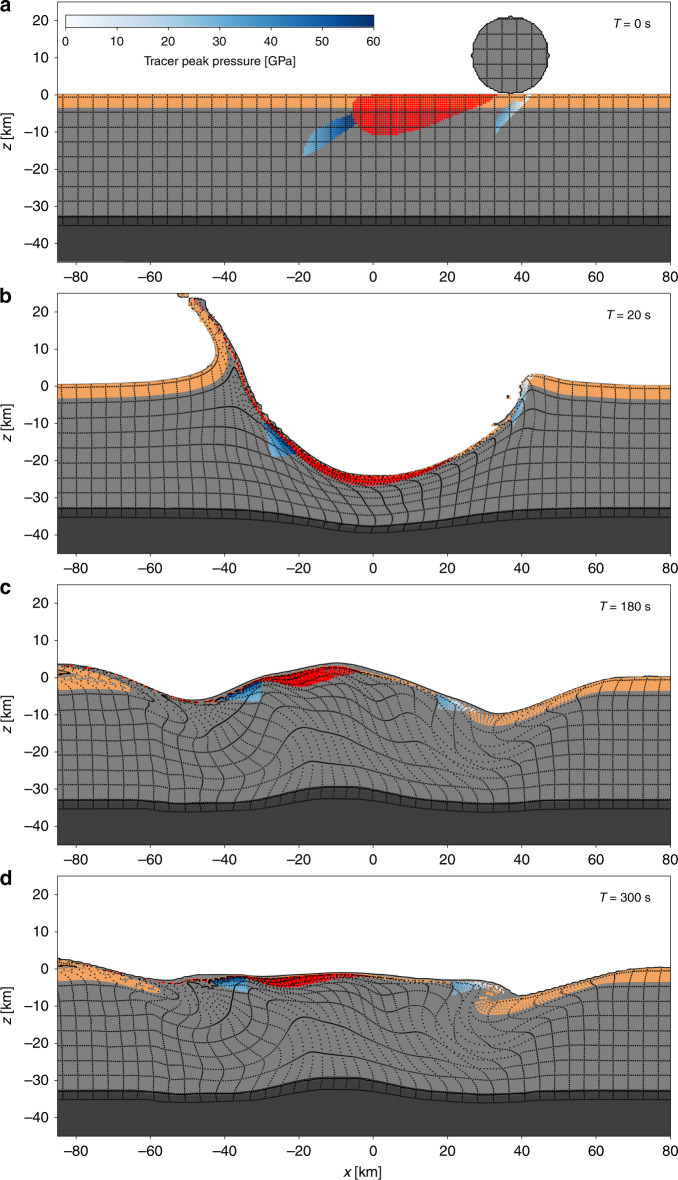
Development of the Chicxulub crater for a $3{0}^{\circ }$ impact. The impact scenario depicted is for a 21-km diameter impactor with a density of 2630 kg m^3^ and a speed of 12 km/s. Evolution of the crater up to 5 min after impact is depicted. Shown are cross-sections through the numerical simulation along the plane of trajectory, with $x=0$ defined at the crater centre (measured at the pre-impact level); the direction of impact is from right to left. Colours and shading of material and tracer particles are the same as Fig. 2.

We performed a series of 3D simulations of impacts that produce a Chicxulub-scale crater, using the iSALE3D shock physics code^18,19^. The simulations assumed a flat, two-layer target comprising crust and mantle and considered four different impact angles ($9{0}^{\circ }$ (vertical), $6{0}^{\circ }$, $4{5}^{\circ }$ and $3{0}^{\circ }$) and two impact speeds (12 and 20 km/s). Further model details are described in the “Methods” section. Our simulations provide insight into crater asymmetries diagnostic of impact angle and trajectory in the absence of any target asymmetry (Figs. 2 and 3, Supplementary Figs. 3–5 and Supplementary Movies 1–4).

In our vertical impact simulation (Supplementary Fig. 3), crater formation is axially symmetric and consistent with previous two-dimensional (2D) numerical simulations that employed an axially symmetric geometry^2,3,17^. Collision of the asteroid with the target surface generates a detached shockwave that propagates symmetrically from the impact site. In the first minute after impact, an excavation flow initiated by the shockwave produces a deep, bowl-shaped cavity, often termed the transient crater. The material flow depresses the crust-mantle boundary beneath the transient crater, uplifts the crust in the transient crater wall and expels the unvaporized portion of the >3-km-thick sedimentary rock sequence from the transient crater as part of the ejecta curtain (Supplementary Fig. 3 and Supplementary Movie 1).

The transient crater is unstable and collapses dramatically to produce a much flatter, broader final crater. In the vertical impact simulation, collapse manifests as uplift of the crater floor and downward and inward collapse of the transient crater rim and a surrounding collar of sedimentary rocks. Floor uplift begins directly beneath the transient crater centre and proceeds vertically upward, overshooting the pre-impact surface to form a large central uplift. At the same time, rim collapse occurs symmetrically at all azimuths, converging towards, and helping to drive up, the central uplift. Finally, the overheightened central uplift of crustal rocks collapses downward and outward, overthrusting the collapsed transient crater rim to form an uplifted ring of crystalline basement, overlying inwardly slumped sedimentary rocks from outside the transient crater. Although the spatial resolution of the numerical simulations is insufficient to resolve the characteristic sharp-peaked topography of the inner ring observed in extraterrestrial peak-ring craters, we are able to identify the position and structure of the material that forms the peak ring in the numerical simulations as a 10-km-wide collar around the central uplift (Supplementary Fig. 2). This model of peak-ring crater formation is supported by geophysical data^20,21^ and recent geological drilling^17^ at Chicxulub, as well as remote-sensing data from the Schrödinger peak-ring crater on the Moon^22^.

**Fig. 4:**
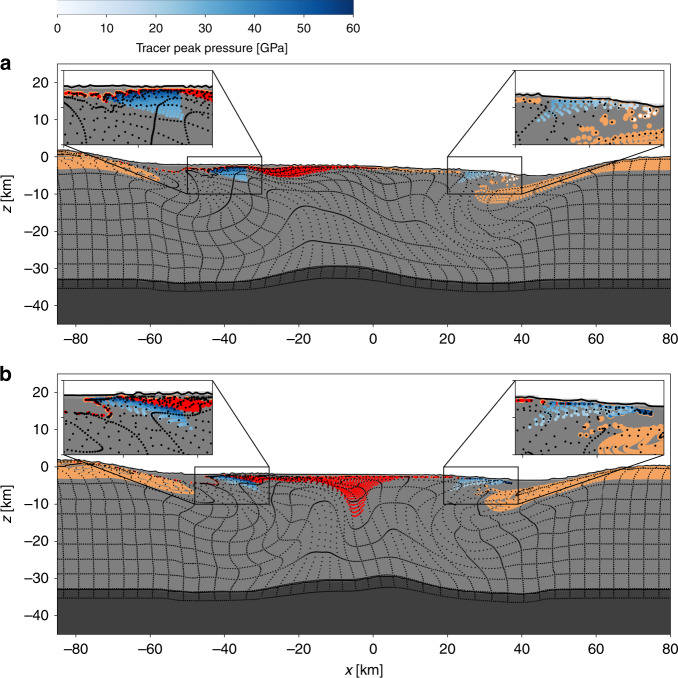
Final simulated Chicxulub crater for a $3{0}^{\circ }$ and $6{0}^{\circ }$ impact angle. Shown are cross-sections, along the plane of trajectory, through the final simulated craters formed by a $3{0}^{\circ }$ (**a**) and $6{0}^{\circ }$ (**b**) impact, with $x=0$ defined at the crater centre (measured at the pre-impact level); the direction of impact is from right to left. Sandy-brown tracers indicate the final position of the upper 3 km of the pre-impact target (sedimentary rock); red tracers indicate the position of melt; tracers with blue–white shading indicate shock pressures of simulated peak-ring materials. The geometric centre of the crater rim defines the coordinate origin ($x=0$); negative $x$-values are downrange.

**Fig. 5:**
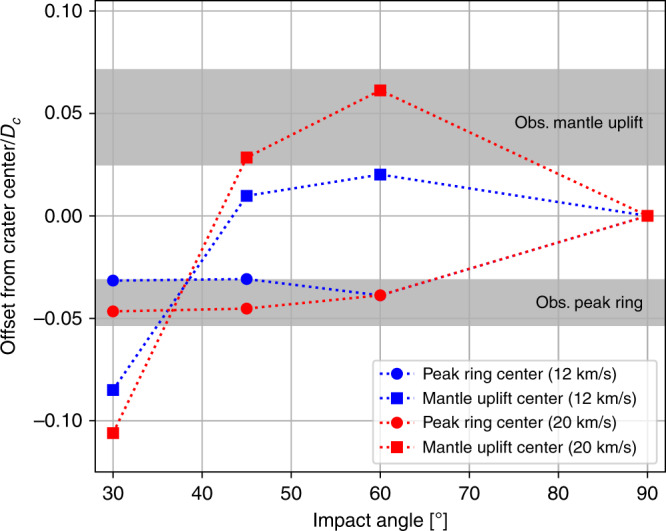
Offset of structural crater features relative to crater centre. The offsets of the centre of mantle uplift (squares) and the centre of the simulated peak ring (circles), relative to the crater centre, are shown as a function of impact angle to the horizontal. Grey bands denote the approximate relative offsets of the peak-ring and mantle-uplift centres at Chicxulub, taking into account the uncertainty in crater diameter and locations of the different features (see Supplementary Fig. 1).

Impacts at progressively shallower angles to the horizontal result in an increasingly asymmetric development of the crater, internally (Figs. 2 and 3, Supplementary Fig.  4; Supplementary Movies 2–4), while the planform of the final impact basin remains approximately circular (Supplementary Table 1; Supplementary Fig. 9). As impact angle decreases, the downrange offset of the crater centre from the impact point increases; less uplift of the transient crater rim occurs in the uprange direction; and more uplift occurs in the downrange direction (Figs. 2 and 3, Supplementary Fig.   4). Relative to the final crater centre, the deepest part of the transient crater (and depressed mantle) also shifts with decreasing impact angle, first to the uprange direction (Fig. 2), then back towards the centre (Supplementary Fig.   4 and Fig. 3). The collapse phase of crater formation is also modified by impact angle. Uplift of the crater floor during crater collapse begins uprange of the crater centre, but has a downrange component such that the central uplift axis is tilted downrange and the centre of the uplift prior to its collapse is downrange of the crater centre (Figs. 2 and 3, Supplementary Fig. 4; Supplementary Movies 2–4). Conversely, downward and outward collapse of the central uplift occurs preferentially in the uprange direction, resulting in enhanced overthrusting of the central uplift on top of transient crater rim in the uprange direction. The net result of the downrange-directed rise and uprange-directed fall of the central uplift is a simulated peak ring with a geometric centre only modestly offset in the downrange direction (Figs. 4 and 5).

The impact simulations shown in Figs. 2 and 3 employ an impact speed of 12 km/s, only slightly larger than the minimum possible speed—Earth’s escape velocity of 11.2 km/s. While these results are likely to be representative of the ~25% of all impacts that occur at speeds below 15 km/s, we also conducted another suite of simulations with a more probable impact speed of 20 km/s (close to Earth’s mean and median asteroid impact speed^23^) to examine the sensitivity of our results to impactor speed. The higher-speed impacts produced similar offsets in mantle-uplift centre and simulated peak-ring centre (compare Figs. 2 and 3 with Supplementary Figs. 6 and 7), and the same trends in offsets with impact angle (Fig. 5).

An important consequence of higher impact speed is enhanced melt production caused by higher shock pressures close to the impact site (e.g., compare Fig. 2a and Supplementary Fig. 6a). The larger melt volume complicates the interpretation of peak-ring structure in the 20 km/s simulations as the dynamics of the melt are not expected to be well captured, given the 500-m spatial resolution of the 3D simulations, and would likely continue long after the simulation end time. Nevertheless, the lateral distribution of the melt material relative to the peak-ring material at the end of the simulations (Supplementary Figs. 8 and 10) suggests that below an impact angle of $4{5}^{\circ }$ there is a high concentration (thick sheet) of surficial melt in the downrange quadrant of the crater, which is likely to hinder or prevent formation of a topographic peak ring at these azimuths. Our results therefore support the idea that horse-shoe shaped peak-ring planforms are indicative of shallow-angle impacts, with the gap in the peak ring diagnostic of the downrange direction^24^.

### Comparison with observations

Asymmetry in crater development produces differences in central crater structure in the uprange and downrange directions. While the centre of the simulated peak ring appears to be consistently offset downrange of the crater centre by ~5% of the crater diameter in the three oblique impacts, the centre of the mantle uplift is offset uprange of the crater centre in the $6{0}^{\circ }$ impact and, to a lesser extent, the $4{5}^{\circ }$ impact; and is offset downrange in the $3{0}^{\circ }$ impact (Fig. 5). This pattern of mantle-uplift offset relative to the final crater rim is a consequence of the corresponding change in the offset of the deepest part of the transient crater relative to the centre of the final crater. Geophysical observations at Chicxulub suggest the peak-ring and mantle-uplift centres are offset in different, approximately opposite directions from the crater centre (Fig. 1). Uncertainty in the precise locations of the centres of the crater, peak ring and mantle uplift (Supplementary Fig. 1), as well as uncertainty in the crater diameter^25^, contribute to an approximate uncertainty of 26% and 48% for the relative offset of the peak ring and mantle uplift, respectively (grey bands in Fig. 5). Comparison of these observations with our simulation results suggests that the observed configuration is most similar to the $6{0}^{\circ }$ impact simulations (or possibly the $4{5}^{\circ }$ impact simulation at 20 km/s; Fig. 5).

Tracer particles that track the history of material in the simulation afford analysis of the provenance of peak-ring materials and their variation with azimuth. The mean depth of origin of peak-ring materials is 10–12 km for the $4{5}^{\circ }$, $6{0}^{\circ }$ and $9{0}^{\circ }$ impacts, only dropping significantly, to ~8 km, in the $3{0}^{\circ }$ impact. In the $3{0}^{\circ }$ scenario a significant fraction of the simulated peak ring originates from the sedimentary sequence in the uprange direction (Fig. 4); the presence of significant amounts of sedimentary material in the simulated peak ring is not consistent with geophysical interpretations or results from Expedition 364^17,26,27^.

We also observe a systematic change in the up/downrange difference in subsurface structure of simulated peak rings with impact angle (Fig. 4). Similar to the situation in a vertical impact, at $6{0}^{\circ }$ the simulated peak ring is formed of overthrusted granitic crustal rocks from the central uplift above down-slumped sedimentary rocks from the transient crater wall, in all directions. However, the sedimentary rocks are deeper and extend farther beneath the simulated peak ring in the uprange direction compared with the downrange direction (Fig. 4). At $4{5}^{\circ }$ and $3{0}^{\circ }$ this difference is more pronounced: on the downrange side of the crater, the inwardly slumped sedimentary rocks do not extend under the simulated peak ring (Fig. 4) owing to enhanced transient crater rim uplift in this direction. This downrange configuration is inconsistent with geophysical interpretations at Chicxulub, which suggest sedimentary slump blocks lie beneath the outer portion of the peak ring at all azimuths offshore^12,25^. However, pre-impact asymmetries in sediment thickness, water depth, particularly in the northeast part of the crater (and potentially in the crust), may also affect structure beneath the peak ring^3^.

A proposed indicator of shallow-angle impact is the truncation of the peak ring in the downrange direction^24^. Our numerical simulations at typical terrestrial impact speeds (20 km/s) are consistent with the production of a gap in the peak ring in the downrange direction for impact angles shallower than 45° (Supplementary Figs. 8 and 10). However, a prominent gap in the Chicxulub peak ring that might indicate a shallow-angle impact is not supported by the geophysical data. The topographic expression of the peak ring is clearly resolved in all radial seismic reflection lines through the offshore portion of the crater^28^ and is particularly prominent in the northwest seismic reflection line Chicx-B^28^, the downrange direction according to shallow-angle impact hypothesis proposed by Schultz and d’Hondt^8^. While the onshore portion of the crater has not been seismically imaged, the annular negative gravity anomaly that has been shown to correlate with peak-ring position offshore is well-pronounced and continuous in this region, with no break that might indicate an abundance of melt or change in the character of the peak ring. The continuity of the geophysical signature of the peak ring therefore also supports a more steeply inclined impact trajectory.

In summary, our numerical simulations of oblique Chicxulub-scale impacts appear to be most consistent with the internal structure of the Chicxulub crater for a steeply inclined impact angle of 45–60° to the horizontal. If the observed asymmetries in the Moho uplift, central uplift and peak ring of the Chicxulub impact structure are attributable to impact trajectory, the implied direction of impact is northeast-to-southwest. This is the opposite direction to that proposed by Hildebrand et al.^11^ based on the offset of the central uplift relative to the crater centre. Our results indicate that uplift of the crater floor occurs in a downrange rather than uprange direction, consistent with numerical simulations of complex crater formation^19^ and geological interpretation of eroded structural uplifts at terrestrial complex craters^9,29^.

Analyses of Venusian craters have not shown a clear link between asymmetries in central crater features and direction of impact. A slight tendency for the peak-ring centre to be offset in the downrange direction was observed, but the results were inconclusive, in part owing to the relatively small number of craters used in the study^30^. The magnitude of the offset (0.03–0.07 D) is, however, consistent with our numerical simulation results. In contrast, there is no correlation between impact trajectory direction and the offset from the crater centre of central peaks in small complex craters^31^. While we did not simulate central peak formation in this work, our results provide a possible explanation for the absence of correlation. At steep angles, uplift of the crater floor initiates uprange of the crater centre, while at shallow angles uplift initiates downrange. If central peaks represent frozen central uplifts, offsets in either uprange or downrange direction might therefore be expected at moderately oblique angles 30–$6{0}^{\circ }$.

### Implications of a steeply inclined Chicxulub impact

Impacts that occur at a steep angle of incidence are more efficient at excavating material and driving open a large cavity in the crust than shallow incidence impacts^5,19^. Our preferred impact angle of ca. 60° is close to the most efficient, vertical scenario, which suggests that previous estimates of impactor kinetic energy based on high-resolution 2D vertical impact simulations^2,17^ do not need to be revised dramatically based on impact angle.

Steeply inclined impacts favour a more symmetric distribution of material ejected from the crater among both proximal and distal ejecta^5^. Asymmetry in the distribution of ejecta was originally used by Schultz and d’Hondt^8^ as an argument for a shallow impact angle towards the northwest. This was based on the observation that both the particle size and layer thickness were relatively large in North American K–Pg sites. Subsequent work has shown that number and size of shocked quartz grains present in the global ejecta layer decreases with distance from Chicxulub, and is independent of azimuth^32–34^. In addition, the 1–3-cm-thick double layer in North America is also observed to the south and southeast of Chicxulub in Colombia^35^ and the Demerara Rise^36^ at equivalent paleodistances from Chicxulub. The global K–Pg boundary layer therefore has a more-or-less symmetric ejecta distribution, consistent with our preferred steep impact angle.

Impact angle has an important influence on the mass of sedimentary target rocks vaporised by the Chicxulub impact^37^. Recent complementary numerical simulations of impact vapour production in oblique impacts using the SOVA shock physics code showed that a trajectory angle of 30–60° constitutes the worst-case scenario for the high-speed ejection of CO_2_ and sulfur by the Chicxulub impact^37^. At this range of impact angles, the ejected mass of CO_2_ is a factor of two-to-three times greater than in a vertical impact and approximately an order of magnitude greater than a very shallow-angle ($1{5}^{\circ }$) scenario^37^. An absence of evaporites in the IODP-ICDP Expedition 364 drill core is consistent with highly efficient vaporisation of sedimentary rocks at Chicxulub^27^. Our simulations therefore suggest that the Chicxulub impact produced a near-symmetric distribution of ejecta and was among the worst-case scenarios for the lethality of the impact by the production of climate-changing gases.

## Methods

### Numerical simulations

The Chicxulub impact was simulated using the iSALE3D shock physics code^18,19^. Tabular equations of state generated using ANEOS^38^ with input parameters for dunite^39^ and granite^40^ were used to describe the thermodynamic response of the mantle and crust, respectively. The impactor was also modelled as a granite sphere, with a density of 2650 kg/m^3^, because of a current limitation of iSALE3D that does not allow for more than one boundary between materials per grid cell. The actual Chicxulub impactor density is not known. Although a carbonaceous chondrite composition has been proposed^41,42^, the bulk porosity of the Chicxulub asteroid prior to impact is undetermined. The Murchison (CM2) carbonaceous chondrite meteorite has a bulk density only 10% less than our impactor density, suggesting that our assumed impactor density is reasonable. Moreover, for a given impactor mass our simulation results are not expected to be sensitive to the assumed impactor density (or other impactor material properties). Material strength was modelled using an approach appropriate for geological materials^43^. The choice of model parameters was based on previous vertical impact simulations using iSALE2D^3,4,17^ and the similar SALEB code^44,45^ and oblique impact simulations of the early stages of the Chicxulub impact^2,46^.

A flat, two-layer target was employed, with a crustal thickness of 33 km. Material number limitations precluded inclusion of a rheologically distinct sedimentary layer in the target; however, Lagrangian tracer particles allowed material at this stratigraphic level to be tracked during the simulation, as well as the peak shock pressure and provenance of peak-ring materials.

We considered four impact trajectory angles, measured relative to the target surface: $9{0}^{\circ }$ (vertical), $6{0}^{\circ }$, $4{5}^{\circ }$ and $3{0}^{\circ }$. Simulations were performed at two impact speeds: 20 and 12 km/s. The slower speed was used for computational expediency and to afford direct comparison of the vertical impact case with previous 2D simulations^3^. The higher impact speed is approximately the average speed that asteroids encounter Earth^23^ and is hence more representative of the likely impact speed of the Chicxulub impact. Impactor diameter was increased with decreasing impact angle (and impact speed) to achieve approximately equivalent final crater diameters (<10% difference). The minimum cell size was 500 m, affording spatial resolutions of 16–21 cells per impactor radius, depending on impact angle and speed.

The impactor size required to produce a Chicxulub-scale crater in our vertical impact simulations with a speed of 12 km/s is slightly larger (14%) than that used in previous 2D simulations^3^. We attribute this discrepancy to a combination of lower spatial resolution in the 3D simulations as well as the absence of a weak sedimentary layer in the upper 3 km of the target. The vertical impact simulations presented here using iSALE3D are consistent with the results of equivalent iSALE2D simulations that employ an equivalent spatial resolution and do not include a separate material layer for the sedimentary rocks.

As with previous simulations of the Chicxulub impact, the acoustic fluidization model^47^ was invoked to explain the temporary dynamic weakening of the target rocks required to facilitate collapse of the transient crater and formation of a final peak-ring crater consistent with geophysical observations^2,3,17^. Acoustic fluidization is a mechanism that reduces the effective resistance to shear deformation of a rock mass subjected to sustained high-frequency pressure fluctuations. In the context of asteroid impacts, initiation of the pressure fluctuations is attributed to the passage of the shockwave; pressure fluctuations subsequently decay in amplitude until they have negligible effect on the internal friction of the rock mass. To ensure consistent application of the acoustic fluidization model for impactors of different size that produce the same size crater, we used fixed acoustic fluidization parameters (viscosity and decay time) in each impact simulation. A full listing of all input parameters is given in Supplementary Table 1.

To analyse the azimuthal variation in peak-ring properties, such as radius and peak shock pressure, it was necessary to identify the Lagrangian tracer particles that track the peak ring. Owing to the relatively low spatial resolution of our 3D simulations, in comparison with previous high-resolution 2D simulations^17^, it was not possible to identify peak-ring materials based on topographic expression within the final crater. Instead, peak-ring material was identified as unmelted (shock pressure $<$60 GPa) material within a 10-km-wide collar of the central uplift, and above the plane defining the base of the central uplift, at the time of maximum uplift ($T\approx 3$ min; Supplementary Fig. 2). The centre of mantle uplift was defined as the $x$-location of the maximum uplift of the mantle material in the simulation. The horizontal peak-ring dimensions were defined based on the final *x*–*y* positions of the peak-ring material tracers (see Supplementary Figs. 9 and 10). Two circles, one inscribing and one circumscribing the peak-ring material tracers, were used to define the inner and outer edge of the simulated peak ring; the centre of the simulated peak ring was calculated as the average of the centres of these two circles. All crater metrics are given in Supplementary Table 1.

## Supplementary information

Supplementary Information

Description of Additional Supplementary Files

Supplementary Movie 1

Supplementary Movie 2

Supplementary Movie 3

Supplementary Movie 4

## Data Availability

Simulation input files, post-processing scripts and output data files are available on Zenodo [10.5281/zenodo.3667833].

## Code availability

At present, the iSALE code is not fully open source. It is distributed via a private GitHub repository on a case-by-case basis to academic users in the impact community, strictly for non-commercial use. Scientists interested in using or developing iSALE should see http://www.isale-code.de for a description of application requirements.
