# Supplementary material for: A steeply-inclined trajectory for the Chicxulub impact
Source: Nat Commun. 2020 May 26;11:1480. doi: 10.1038/s41467-020-15269-x (PMC7251121; doi:10.1038/s41467-020-15269-x)
Supplement: Supplementary file 1 — Supplementary Information [file 41467_2020_15269_MOESM1_ESM.pdf]

Supplementary Information for:

A steeply-inclined trajectory for the Chicxulub impact

G. S. Collins *et al*.

Supplementary Tables

Supplementary Table 1 Numerical model parameters and crater metrics

Parameter definition	Crust & Impactor	Mantle
Reference density [kg m^{-3}]	2630	3310
Poisson's ratio	0.3	0.25
Melt temperature (zero pressure)[1] [K]	1673	1373
Simon approximation parameter (a)[1] [GPa]	6	1.52
Simon approximation parameter (c)[1]	3	4.05
Thermal softening parameter (ξ)[2]	1.2	1.2
Intact strength (zero pressure)[2] [MPa]	10	10
Intact coefficient of friction[2]	2	1.2
Intact strength limit[2] [GPa]	2.5	3.5
Damaged strength (zero pressure)[2] [MPa]	0.01	0.01
Damaged coefficient of friction[2]	0.6	0.6
Damaged strength limit[2] [GPa]	2.5	3.5

Minimum failure strain at low pressure (ϵ_{fmin})[3]	10^{-4}
Slope of failure strain vs. pressure ($k_{p\epsilon}$)[3] [Pa^{-1}]	10^{-11}
Transition pressure for failure strain (p_{ϵ})[3] [MPa]	300
Vibrational particle velocity as a fraction of particle velocity[4]	0.1
Maximum vibrational particle velocity[4] [m s^{-1}]	200
Time after which no new vibrations are generated[4] [s]	16
Decay time of acoustic vibrations[4] [s]	185
Kinematic viscosity of acoustically fluidized material[4] [m^2 s^{-1}]	320,000

Impact angle to horizontal [°]	90	60	45	30
Impactor radius (for 12 km/s impact speed) [km]	8	8.5	9.	10.5
Impactor radius (for 20 km/s impact speed) [km]	6	6.5	7	8
Crater dimensions for 12 km/s impact speed				
Apparent crossrange crater diameter (pre-impact level) [km]	130	134	133	133
Apparent alongrange crater diameter (pre-impact level) [km]	130	138	142	152
Peak ring inner diameter [km]	50	52	56	57
Peak ring outer diameter [km]	84	82	80	84
Peak ring diameter [km]	67	67	68	71
Transient crater diameter [km]	72	80	83	91
Mantle uplift centre offset (+ve uprange) [km]	0	2.7	1.3	-11.3
Inner peak ring centre offset (+ve uprange) [km]	0	-6.0	-4.9	-5.8
Outer peak ring centre offset (+ve uprange) [km]	0	-4.4	-3.2	-2.6
Peak ring centre offset (+ve uprange) [km]	0	-5.2	-4.1	-4.2
Crater dimensions for 20 km/s impact speed				
Apparent crossrange crater diameter (pre-impact level) [km]	120	129	137	133
Apparent alongrange crater diameter (pre-impact level) [km]	120	129	132	145
Peak ring inner diameter [km]	45	46	51	56
Peak ring outer diameter [km]	89	89	85	80
Peak ring diameter [km]	67	68	68	68
Transient crater diameter [km]	74	81	84	85
Mantle uplift centre offset (+ve uprange) [km]	0	7.9	3.9	-14.1
Inner peak ring centre offset (+ve uprange) [km]	0	-3.5	-5.3	-7.8

Outer peak ring centre offset (+ve uprange) [km]	0	-6.5	-7.2	-4.7
Peak ring centre offset (+ve uprange) [km]	0	-5.0	-6.2	-6.2

1. Melt curve parameters [1]
2. Strength model parameters [2]
3. Failure strain model: $\epsilon_f = \max(\epsilon_{fmin}, k_{p\epsilon}[p - p_\epsilon])$ [3]
4. Acoustic fluidization model parameters [4]; note the corresponding scaling constants (γ_η, γ_β) vary depending on the impactor radius.

Supplementary Figures

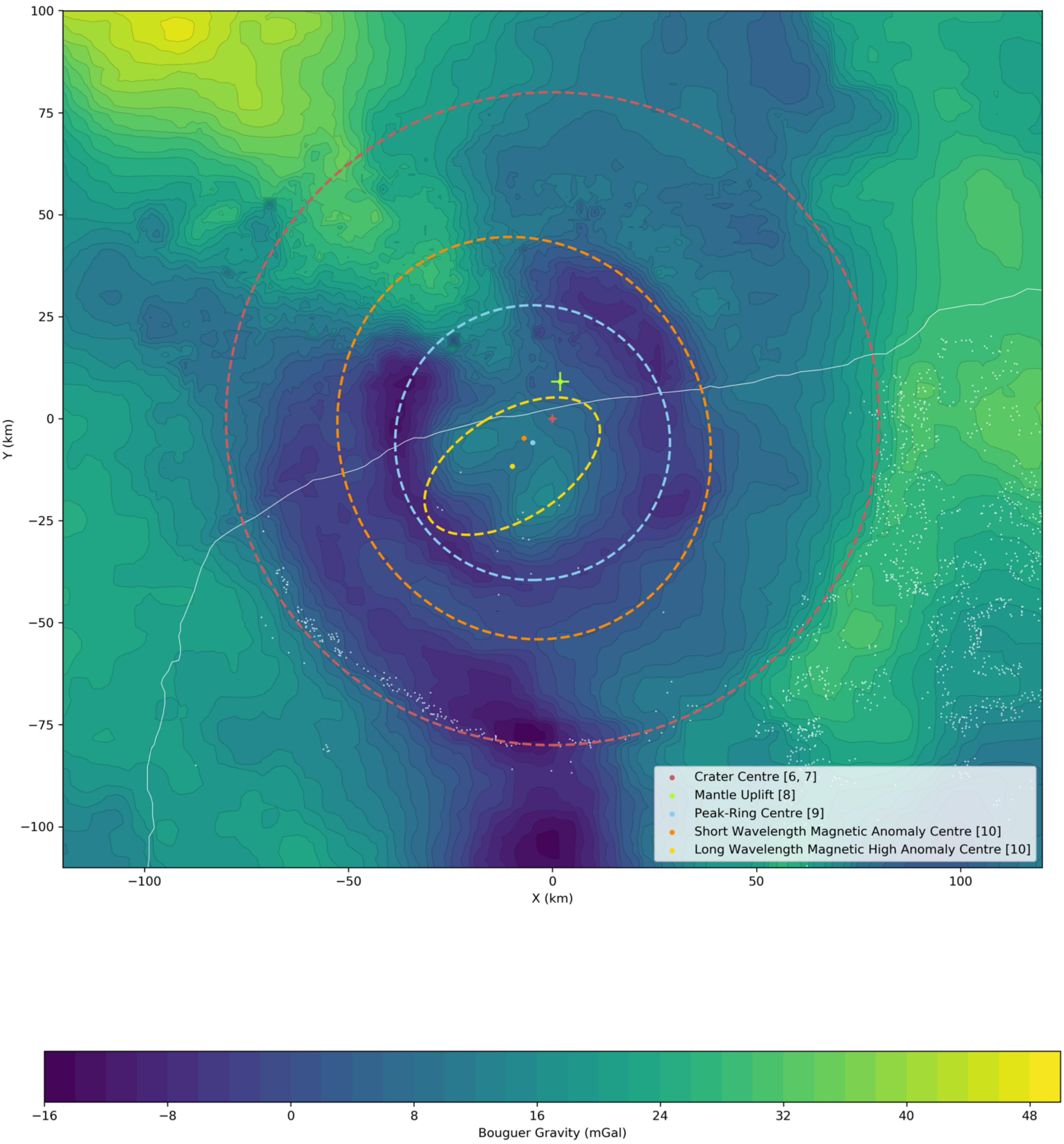

Supplementary Figure 1: Compilation of geophysical anomalies of the Chicxulub impact structure on a Bouguer Gravity map of the crater vicinity (gravity data courtesy of A. R. Hildebrand and M. Pilkington). The coordinate system has been transformed to a cartesian grid, with the origin at the crater centre, following the data transformation of Ref.[5] Red circle, red dot and 1-km errorbars indicate the crater margin, centre and uncertainty, respectively, based on the outer margin of the gravity low also associated with a maximum in horizontal gravity gradient and the ring of cenotes[6,7] (Fig. 1). Green dot with 2-km errorbars indicates the location of the mantle uplift centre[8], which is offset 9.3 km NNE from the crater centre. Blue circle and dot indicate the position and approximate diameter of the peak ring, respectively, based on the annular gravity low inside the crater[9], which is offset approximately 7.6 km SW of the crater centre. Orange ellipse and dot indicate the outline and centre of the short wavelength magnetic anomaly, interpreted to represent the extent of the melt sheet[10]. Note the slight elongation in the NNW-SSE direction and the offset of the centre to the SW of the crater centre. The yellow ellipse and dot indicate the outline and centre of the long-wavelength magnetic anomaly, interpreted to represent uplift of mid-crustal rocks[10]. Note the elongation and offset of this anomaly to the SW.

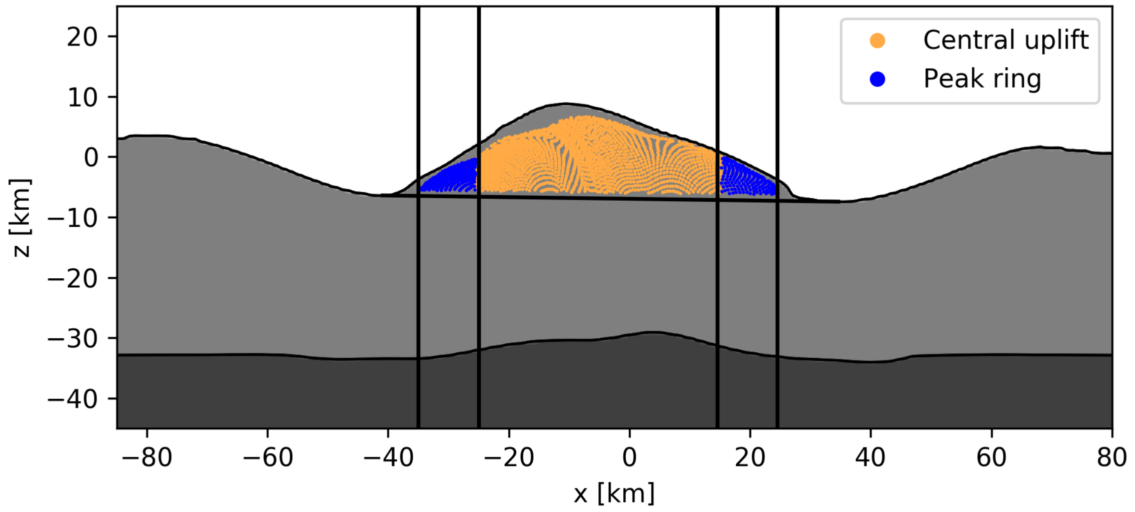

Supplementary Figure 2 Example of how Lagrangian tracer particles were used to identify peak ring material. Peak ring material was identified as those tracers within a 10-km wide collar of the central uplift (blue tracers), and above the plane defining the base of the central uplift (green and blue tracers), at the time of maximum uplift (200 s in the example shown).

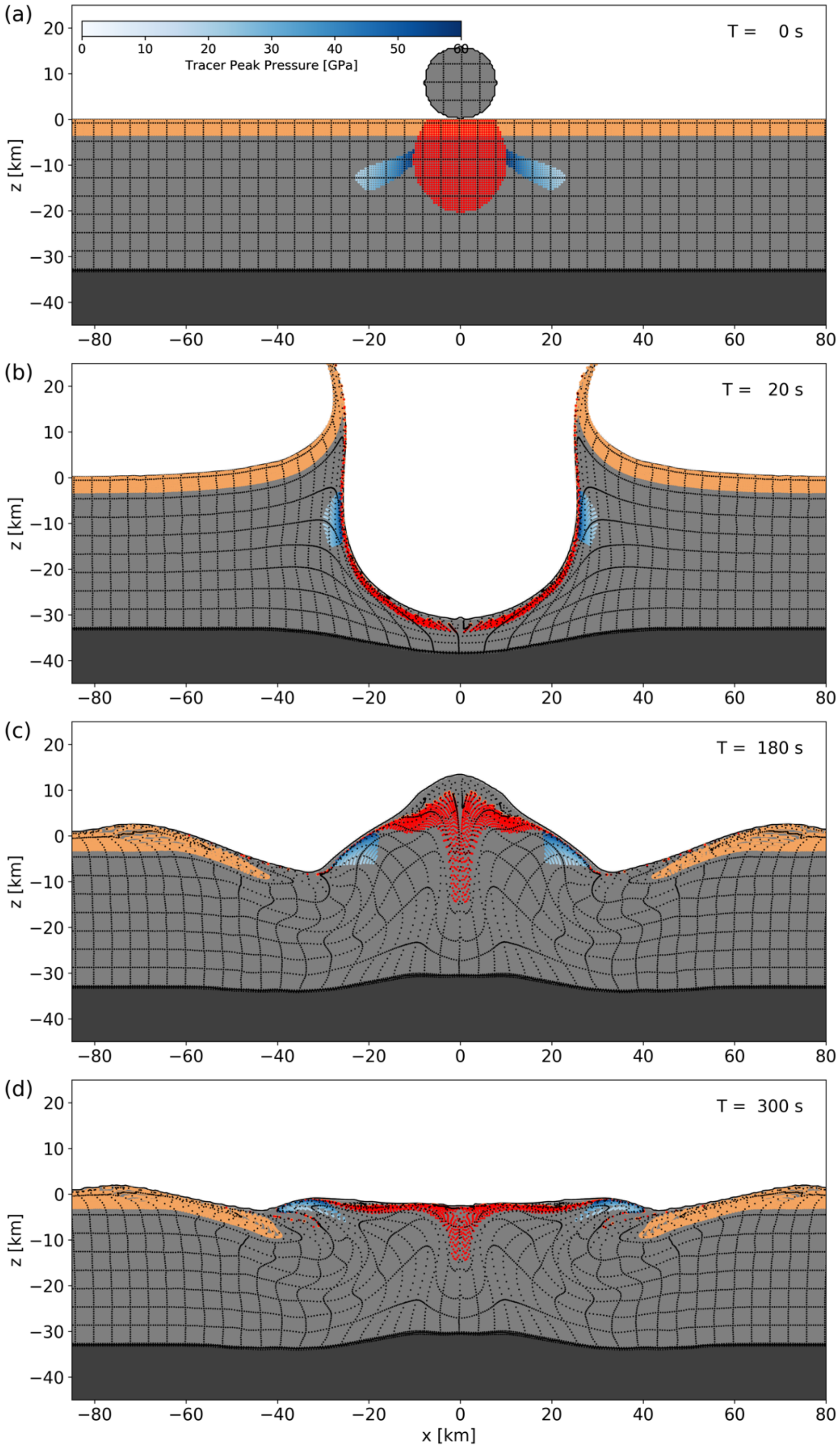

Supplementary Figure 3: Development of the Chicxulub crater for a 90° impact during the first 5 minutes. The scenario depicted is for a 16-km diameter impactor with a density of 2650 kg/m^3 and a speed of 12 km/s. Shown is a cross-section through the numerical simulation along the plane of trajectory, with x=0 defined at the crater centre (measured at the preimpact level). The upper 3-km of the preimpact target, corresponding to the average thickness of sedimentary rocks at Chicxulub, is tracked by tracer particles (sandy brown). Deformation in the crust (grey) and upper mantle (dark grey) is depicted by a grid of tracer particles (black). Tracer particles within the peak-ring material are highlighted based on the peak shock pressure recorded (white-blue colour scale); melted target material is highlighted in red.

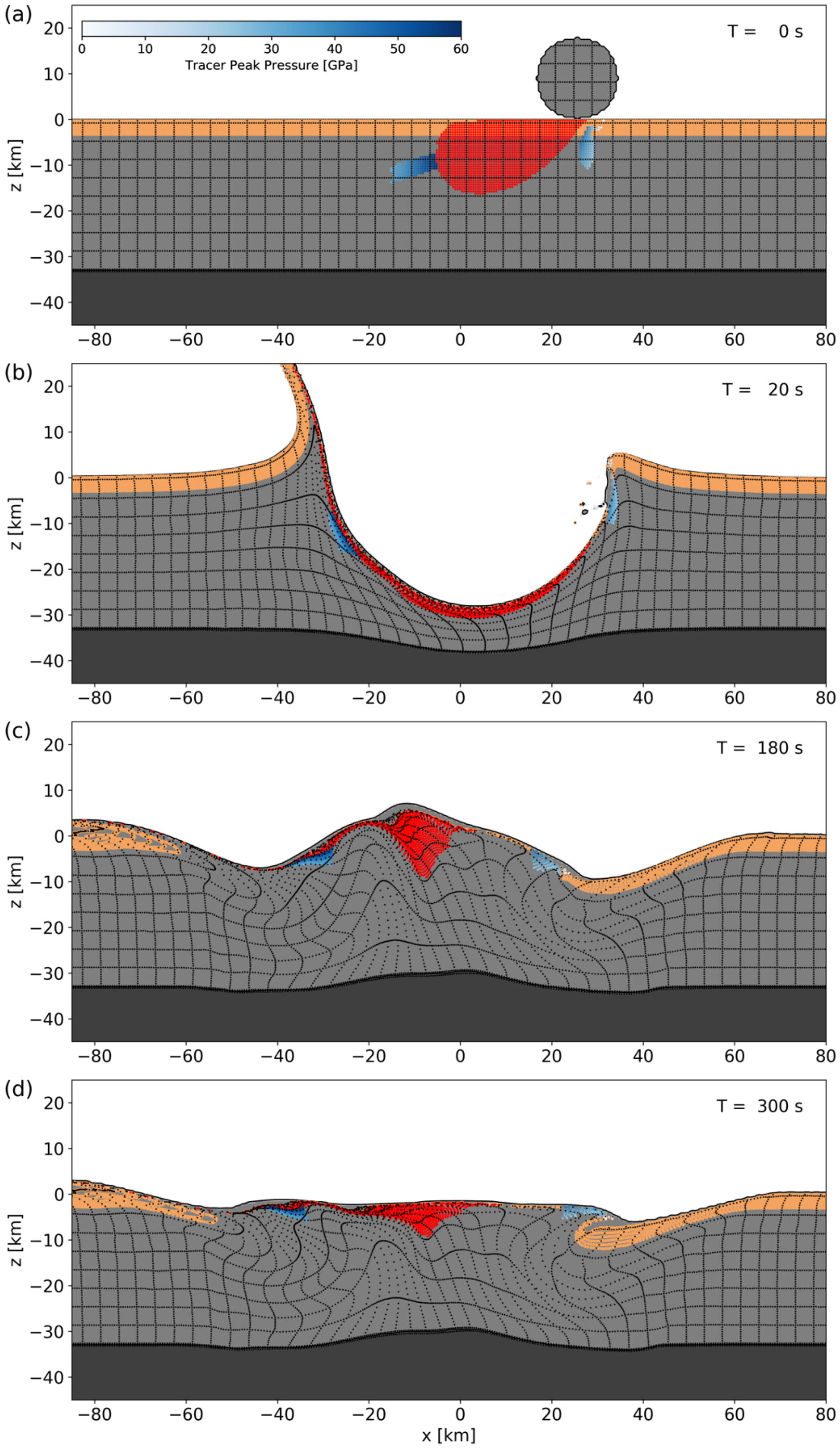

Supplementary Figure 4: Development of the Chicxulub crater for a 45° impact during the first 5 minutes. The scenario depicted is for a 18-km diameter impactor with a density of 2650 kg/m^3 and a speed of 12 km/s. Shown is a cross-section through the numerical simulation along the plane of trajectory, with x=0 defined at the crater centre (measured at the preimpact level) and the direction of impact is from right to left. Colours and shading of material and tracer particles is the same as Figure S1.

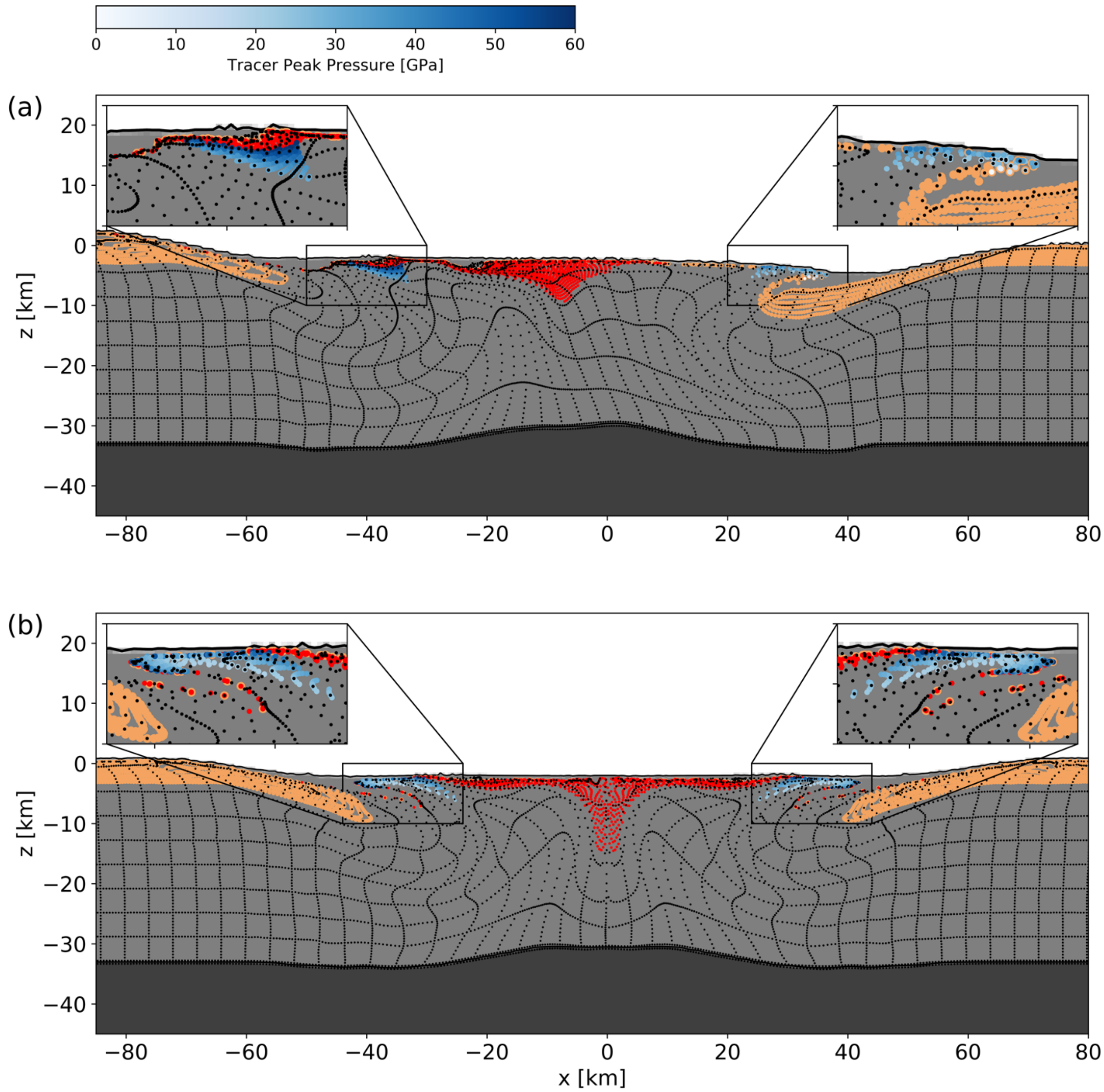

Supplementary Figure 5: Cross-sections of the final simulated Chicxulub crater, in the plane of the impact trajectory, for a 45° (a) and 90° (b) impact angle (to the target plane) for an impact speed of 12 km/s. Impact direction is right to left in (a). Sandy-brown tracers indicate the final position of the upper 3-km of the preimpact target (sediments); red tracers indicate the position of melt; tracers with blue-white shading indicate shock pressures of peak-ring materials. The geometric centre of the crater rim defines the coordinate origin x = 0; negative x-values are downrange in (a).

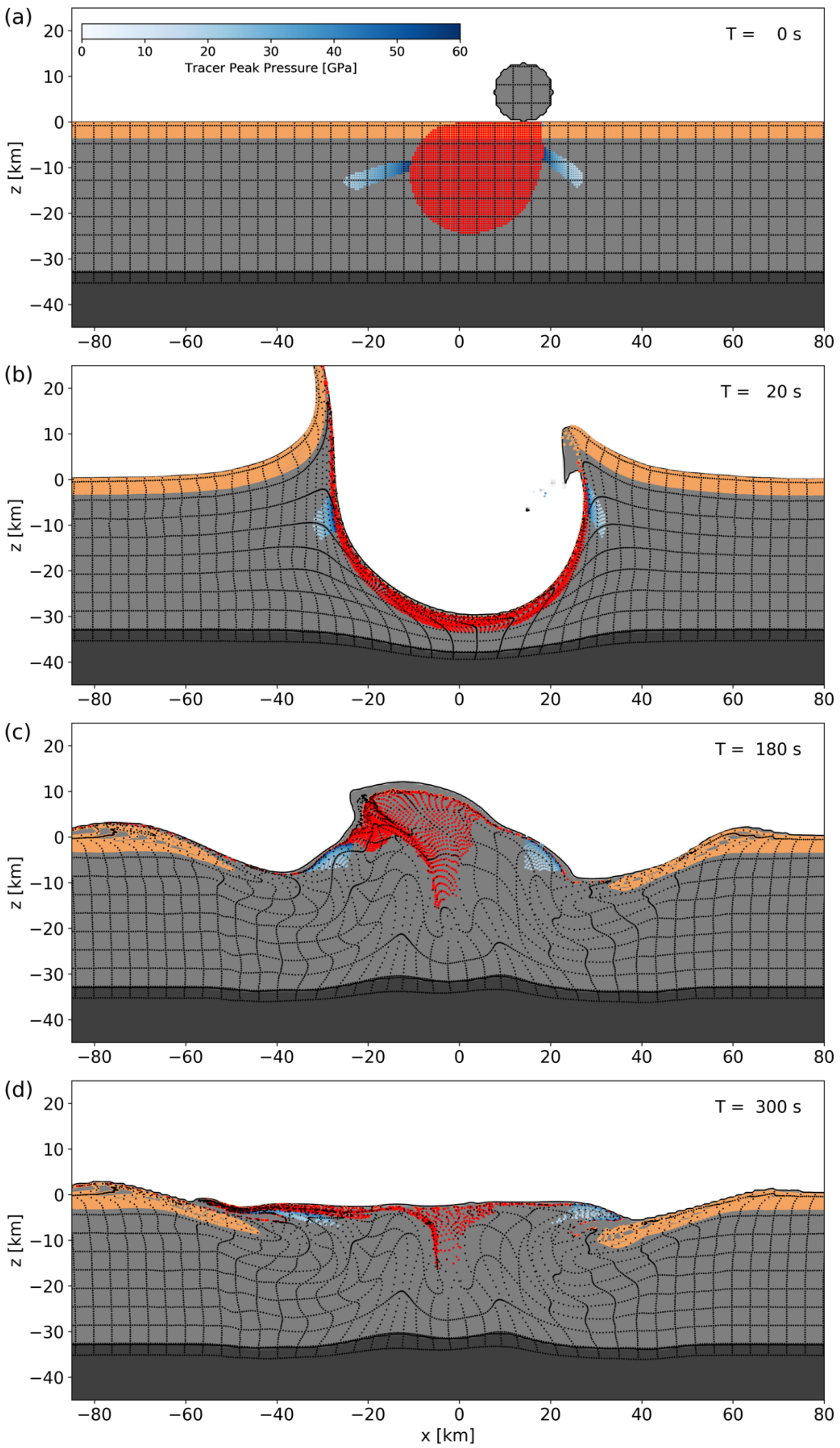

Supplementary Figure 6: Development of the Chicxulub crater for a 60° impact during the first 5 minutes. The scenario depicted is for a 13-km diameter impactor with a density of 2650 kg/m^3 and a speed of 20 km/s. Shown is a cross-section through the numerical simulation along the plane of trajectory, with x = 0 defined at the crater centre (measured at the preimpact level) and the direction of impact is from right to left. Colours and shading of material and tracer particles is the same as Figure S1.

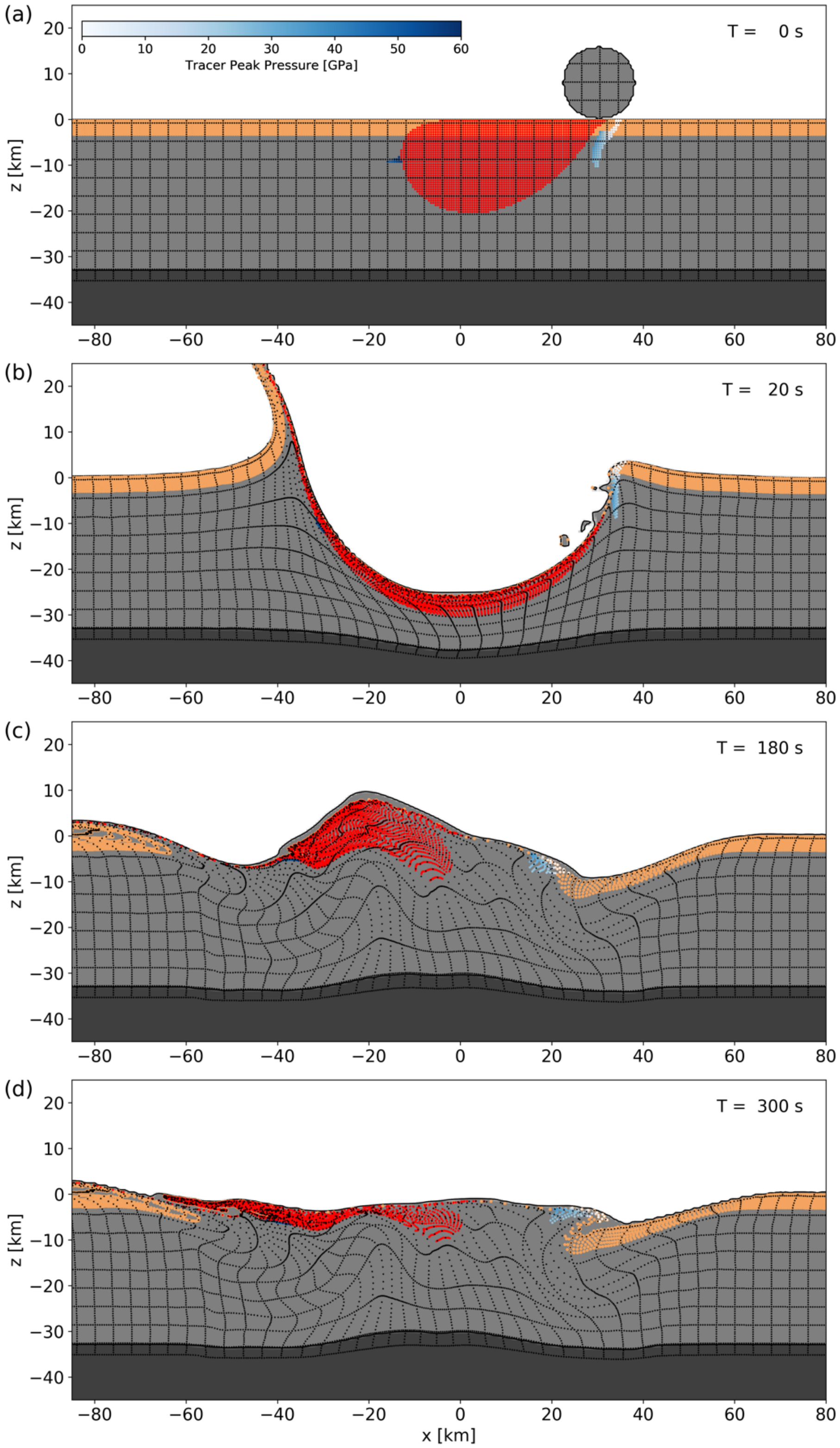

Supplementary Figure 7: Development of the Chicxulub crater for a 30° impact during the first 5 minutes. The scenario depicted is for a 16-km diameter impactor with a density of 2650 kg/m^3 and a speed of 20 km/s. Shown is a cross-section through the numerical simulation along the plane of trajectory, with x = 0 defined at the crater centre (measured at the preimpact level) and the direction of impact is from right to left. Colours and shading of material and tracer particles is the same as Figure S1.

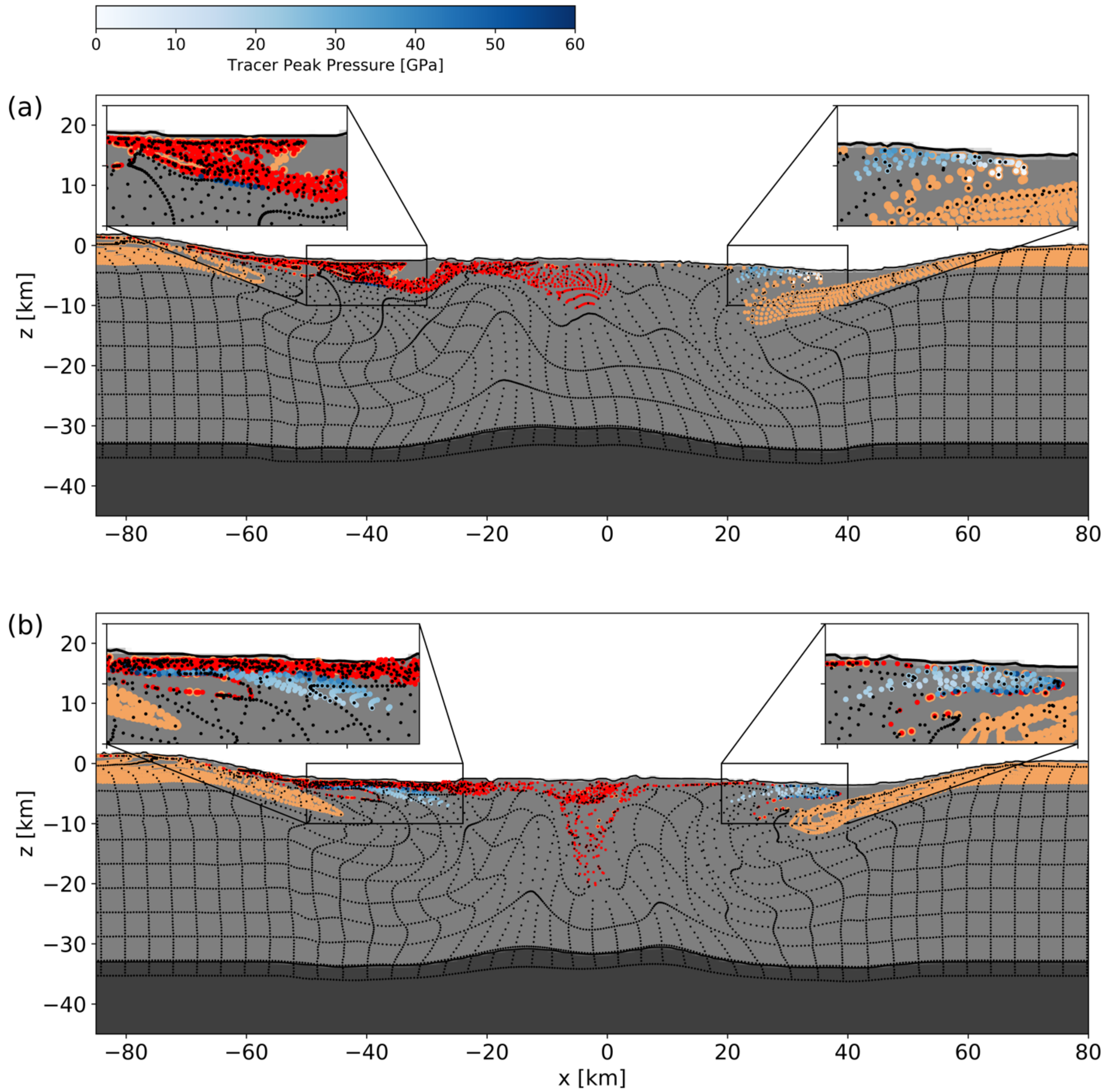

Supplementary Figure 8: Cross-sections of the final simulated Chicxulub crater, in the plane of the impact trajectory, for a 30° (a) and 60° (b) impact angle (to the target plane) for an impact speed of 20 km/s. Impact direction is right to left in (a). Sandy-brown tracers indicate the final position of the upper 3-km of the preimpact target (sediments); red tracers indicate the position of melt; tracers with blue-white shading indicate shock pressures of peak-ring materials. The geometric centre of the crater rim defines the coordinate origin x = 0; negative x-values are downrange in (a).

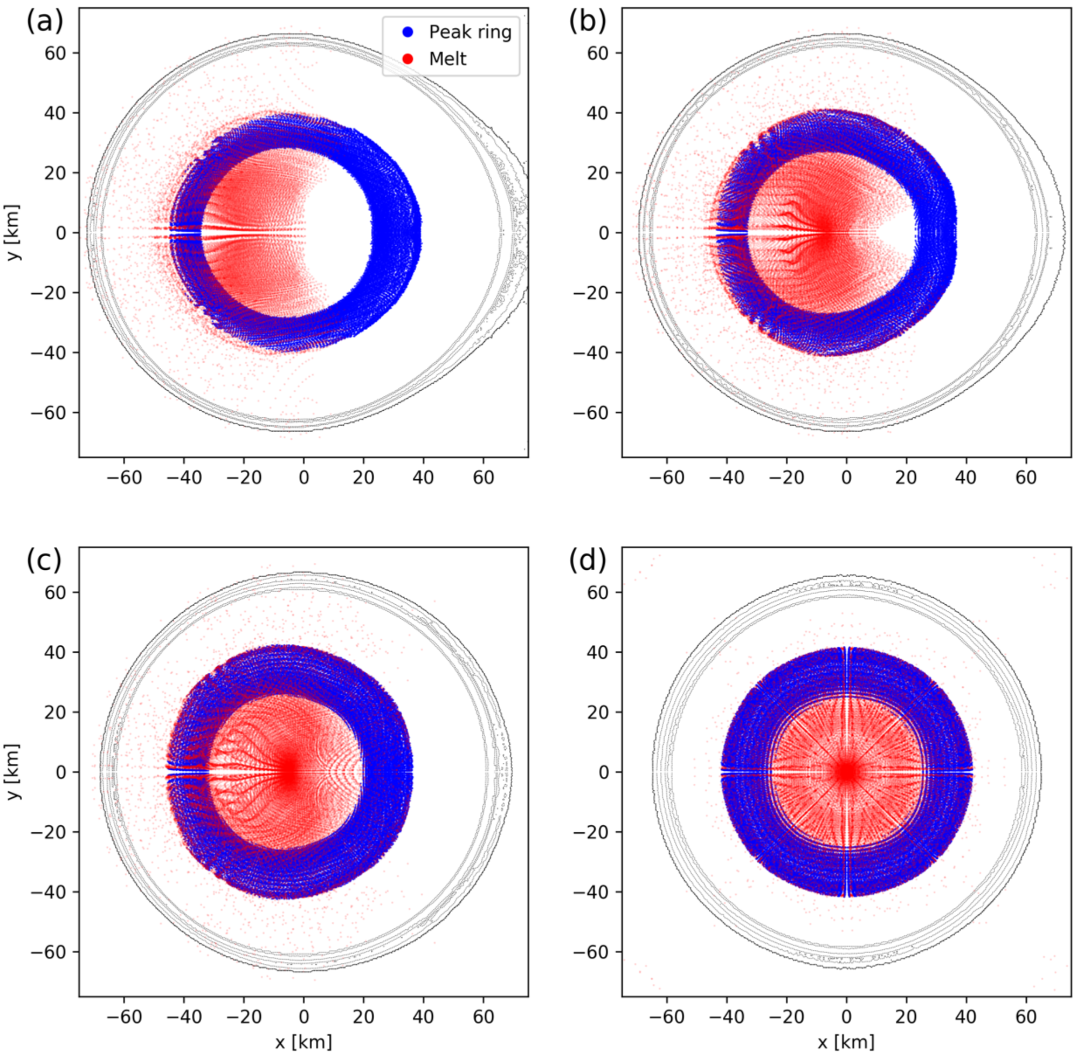

Supplementary Figure 9: Plan view of the crater outline (as defined by the –1 to 0 elevation contours) and the spatial distribution of peak-ring material tracers (blue) and melt tracers (red) at the end of the simulation for scenarios with an impact speed of 12 km/s and an impact angle of (a) 30°, (b) 45°, (c) 60°, (d) 90°. The geometric centre of the crater rim defines the coordinate origin x = 0; negative x-values are downrange in the oblique cases.

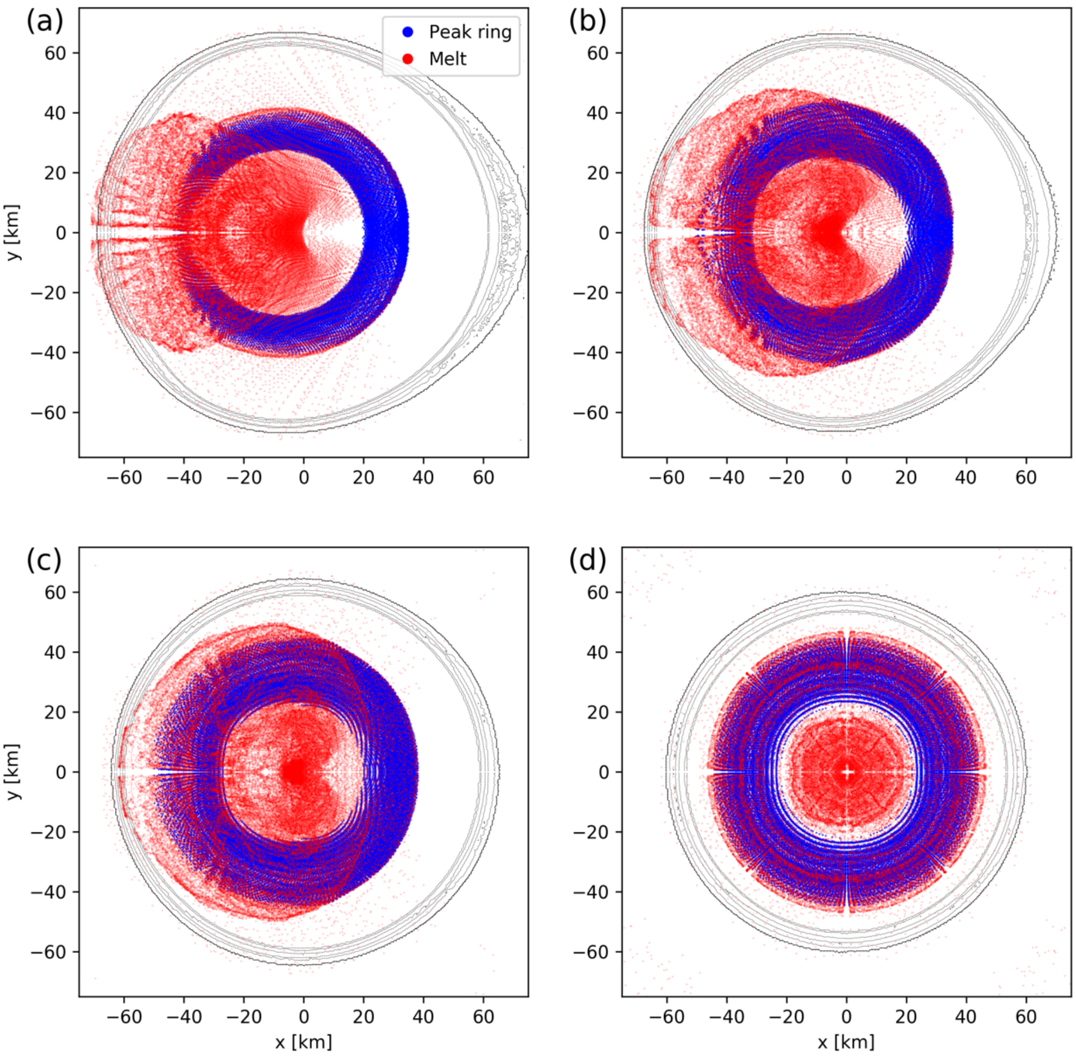

Supplementary Figure 10: Plan view of the crater outline (as defined by the –1 to 0 elevation contours) and the spatial distribution of peak-ring material tracers (blue) and melt tracers (red) at the end of the simulation for scenarios with an impact speed of 20 km/s and an impact angle of (a) 30°, (b) 45°, (c) 60°, (d) 90°. The geometric centre of the crater rim defines the coordinate origin x = 0; negative x-values are downrange in the oblique cases. At this higher impact speed, the melt volume is greater and there are more melt tracers, which are concentrated downrange. In the 30° impact angle scenario (a), a thick layer of melt overlies the peak-ring material tracers in the downrange direction which is expected to result in no topographic expression of the peak ring at this azimuth. In such scenarios, the morphology of the final crater is expected to exhibit a horse shoe shaped peak ring, with a gap in the down range direction.

Supplementary References

1. Wünnemann, K., Collins, G. S. & Osinski, G. R. Numerical modelling of impact melt production in porous rocks. *Earth and Planetary Science Letters* **269**, 530–539 (2008).
2. Collins, G. S., Melosh, H. J. & Ivanov, B. A. Modeling damage and deformation in impact simulations. *Meteoritics & Planetary Science* **39**, 217–231 (2004).
3. Ivanov, B. A., Melosh, H. J. & Pierazzo, E. Basin-forming impacts: Reconnaissance modeling. *Geological Society of America Special Papers* **465**, 29–49 (2010).
4. Wünnemann, K. & Ivanov, B. A. Numerical modelling of the impact crater depth–diameter dependence in an acoustically fluidized target. *Planetary and Space Science* **51**, 831–845 (2003).
5. Vermeesch, P. M., Morgan, J. V., Christeson, G. L., Barton, P. J. & Surendra, A. Three-dimensional joint inversion of traveltime and gravity data across the Chicxulub impact crater. *Journal of Geophysical Research: Solid Earth* **114**, (2009).
6. Hildebrand, A. R., Pilkington, M., Connors, M., Ortiz-Aleman, C. & Chavez, R. E. Size and structure of the Chicxulub crater revealed by horizontal gravity gradients and cenotes. *Nature* **376**, 415–417 (1995).
7. Morgan, J. *et al.* Size and morphology of the Chicxulub impact crater. *Nature* **390**, 472–476 (1997).
8. Christeson, G. L. *et al.* Mantle deformation beneath the Chicxulub impact crater. *Earth and Planetary Science Letters* **284**, 249–257 (2009).
9. Hildebrand, A. R. *et al.* Mapping Chicxulub crater structure with overlapping gravity and seismic surveys. in *29th Lunar and Planetary Science Conference* Abstract #1821 (1998).
10. Pilkington, M. & Hildebrand, A. R. Three-dimensional magnetic imaging of the Chicxulub Crater. *Journal of Geophysical Research: Solid Earth* **105**, 23479–23491 (2000).
