# Supplementary material for: A steeply-inclined trajectory for the Chicxulub impact
Source: Nat Commun. 2020 May 26;11:1480. doi: 10.1038/s41467-020-15269-x (PMC7251121; doi:10.1038/s41467-020-15269-x)
Supplement: Supplementary file 2 — Description of Additional Supplementary Files [file 41467_2020_15269_MOESM2_ESM.pdf]

## Description of Supplementary Files

**File name:** Supplementary Movie 1
**Description**: Development of the Chicxulub crater for a 90° impact. The scenario depicted is for a 8-km radius impactor with a density of 2650 kg/$m^3$ and a speed of 12 km/s. Shown is a cross-section through the numerical simulation along the plane of trajectory, with x=0 defined at the crater centre (measured at the preimpact level) for ten minutes after impact. The upper 3-km of the preimpact target, corresponding to the average thickness of sedimentary rocks at Chicxulub, is tracked by tracer particles (sandy brown). Deformation in the crust (grey) and upper mantle (dark grey) is depicted by a grid of tracer particles (black). Tracer particles within the peak-ring material are highlighted based on the peak shock pressure recorded (white-blue colour scale); melted target material is highlighted in red.

**File name:** Supplementary Movie 2
**Description**: Development of the Chicxulub crater for a 60° impact. The scenario depicted is for a 8.5-km radius impactor with a density of 2650 kg/$m^3$ and a speed of 12 km/s. Shown is a cross-section through the numerical simulation along the plane of trajectory for ten minutes after impact, with x=0 defined at the crater centre (measured at the preimpact level) and the direction of impact is from right to left. Colours and shading of material and tracer particles is the same as Supplementary Movie 1.

**File name:** Supplementary Movie 3
**Description**: Development of the Chicxulub crater for a 45° impact. The scenario depicted is for a 9-km radius impactor with a density of 2650 kg/$m^3$ and a speed of 12 km/s. Shown is a cross-section through the numerical simulation along the plane of trajectory for ten minutes after impact, with x=0 defined at the crater centre (measured at the preimpact level) and the direction of impact is from right to left. Colours and shading of material and tracer particles is the same as Supplementary Movie 1.

**File name:** Supplementary Movie 4
**Description**: Development of the Chicxulub crater for a 30° impact. The scenario depicted is for a 10.5-km radius impactor with a density of 2650 kg/$m^3$ and a speed of 12 km/s. Shown is a cross-section through the numerical simulation along the plane of trajectory for ten minutes after impact, with x=0 defined at the crater centre (measured at the preimpact level) and the direction of impact is from right to left. Colours and shading of material and tracer particles is the same as Supplementary Movie 1.
